# Beta arrestin‐related signalling axes are influenced by dexamethasone and metformin in vascular smooth muscle cells cultured in high glucose condition

**DOI:** 10.1002/edm2.465

**Published:** 2023-12-15

**Authors:** Ali Akbar Soleimani, Nafiseh Shokri, Mohammad Elahimanesh, Payam Mohammadi, Najmeh Parvaz, Masoomeh Bakhshandeh, Mohammad Najafi

**Affiliations:** ^1^ Department of Clinical Biochemistry, Faculty of Medicine Tehran University of Medical Sciences Tehran Iran; ^2^ Department of Clinical Biochemistry, Faculty of Medicine Iran University of Medical Sciences Tehran Iran; ^3^ Cellular and Molecular Research Center Iran University of Medical Sciences Tehran Iran; ^4^ Microbial Biotechnology Center Iran University of Medical Sciences Tehran Iran

**Keywords:** BARR2, dexamethasone, high‐glucose, metformin, VSMC

## Abstract

**Background:**

Metformin (Met) and dexamethasone (Dexa) are known to reduce blood sugar levels and anti‐inflammatory effects, respectively. Based on the acceleration of atherosclerosis process in diabetes, the β‐arrestin 2 (BARR2) gene and protein expression levels were evaluated in vascular smooth muscle cells (VSMCs) treated with Met and Dexa in high glucose conditions in this study.

**Methods and Materials:**

Human VSMCs were cultured in Dulbecco's Modified Eagle Medium/Nutrient Mixture F‐12 (DMEM‐F12) medium and, were treated with different values of Met (1 mM, 5 mM and 7 mM) and Dexa (10^−7^ M, 10^−6^ M and 10^−5^ M) in 24‐ and 48‐h periods. The BARR2 gene and protein expression levels were identified with RT‐qPCR and western blotting techniques, respectively. The signalling axes were predicted from gene network made using Cytoscape software and, were annotated with Gene Ontology.

**Results:**

The BARR2 gene and protein expression levels reduced in VSMCs treated with Dexa and Met after 24‐ and 48‐h periods. These results were more changed after 48 h. Furthermore, many BARR2‐related signalling axes were found from the network genes.

**Conclusion:**

Met and Dexa suppressed the BARR2 protein and gene expression levels in the VSMCs. Moreover, the gene network suggested some the cellular signalling axes related to BARR2 that may be affected by Met and Dexa.

## Graphical abstract

The study results showed that metformin (Met) similar to dexamethasone (Dexa) suppresses the β‐arrestin 2 (BARR2) protein and gene expression levels in the vascular smooth muscle cells (VSMCs). Furthermore, the gene network data suggested that BARR2‐related signalling axes might affect many cellular signalling pathways by Met and Dexa. However, more studies are needed to find the roles of Met and Dexa in these signalling pathways.

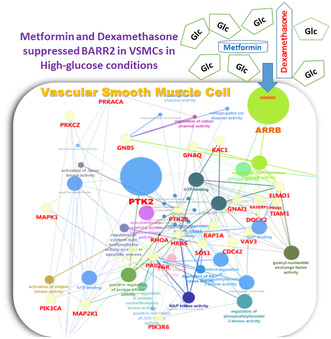

## 1 INTRODUCTION

Atherosclerotic cardiovascular disease is known as one of the main causes of death in the world.^1^ Some risk factors such as dyslipidaemia, hyperglycaemia, hypertension, genetics and, lifestyle reported to be involved in the development and progression of atherosclerosis process.^1^, ^2^ From the point of molecular and cellular pathogenesis, atherosclerosis is known as a progressive event followed due to monocyte internalization through adhesion molecules, cellular polarization, formation and development of plaque within subendothelium space.^3^ Monocytes differentiate into macrophages and convert to foam cells due to scavenging the lipid particles such as Ox‐LDL.^4^ The vascular smooth muscle cells (VSMCs) also play a key role in the development of atherosclerosis. These cells migrate into intima layer by stimulators such as interleukins, tumour necrosis factor‐α and platelet‐derived growth factor.^5^, ^6^ It is reported that hyperglycaemia promotes endothelial dysfunction and induces the VSMC migration and proliferation through the NF‐kB signalling pathway.^7^, ^8^, ^9^ It is also reported that β‐arrestin 2 (BARR2), as a modulator of 7TM GPCRs, by activating MEK/ERK signalling pathway in VSMCs promotes the atherosclerosis process.^10^ Metformin (Met) is an anti‐diabetic drug that through AMP‐activated protein kinase (AMPK) decreases the VSMC migration and proliferation and ameliorates atherosclerosis under hyperglycaemic conditions.^11^, ^12^, ^13^ Dexamethasone (Dexa) and other corticosteroids are also well recognized to affect immunological events^14^ so that these drugs are suggested for the treatment of atherosclerosis.^15^, ^16^ In addition, some studies showed that Dexa suppresses the migration and proliferation of VSMCs.^14^, ^17^, ^18^

FAK (PTK2), as a key gene in the chemokine signalling pathway, has recently reported to be involved in the proliferation and migration of VSMCs treated with Met.^19^ BARR2 is also a nuclear internalized protein in the chemokine signalling pathway so that we proposed Met and Dexa might affect its function in VSMCs. Based on the above descriptions, the aim of this study was to investigate the BARR2 gene and protein expression levels in VSMCs treated with Met and Dexa. In addition, the enriched gene networks were predicted to understand and explain the roles of BARR2 in the cellular signalling axes.

## 2 MATERIALS AND METHODS

### 2.1 Cell culture

VSMCs (National Cell Bank, Pasteur Institute, C591) were cultured in DMEM‐F12 containing FBS 10% (Gibco, Thermo Fisher Scientific‐US) and Pen‐strep 1% (Sigma‐Aldrich Co., St Louis, MO, USA) (5% CO2, 37°C). These cells were studied in 24 and 48‐h periods including: (A) Normal group, (B) High glucose (HG) group (25 mM),^13^, ^19^ (C) HG and Met groups (1 mM, 5 mM and 7 mM),^19^ (D) HG and Dexa groups (10^−7^ M, 10^−6^ M and 10^−5^ M). The VSMCs were exposed to Met and Dexa during the studied periods (24 and 48 h). The cellular and molecular tests were repeated three times (*n* = 3).

### 2.2 Gene networking

The work was followed on the results of FAK suppression in the Met‐treated VSMCs.^19^ Similar to FAK (PTK2), BARR (ARRB), as an important central gene, was proposed to transduce the cellular signals from the upstream of chemokine signalling pathway (KEGG, hsa 04062). A primary gene network including the BARR and FAK were visualized by transferring the components of chemokine signalling pathway into Cytoscape (version 3.9.1) software using KEGGscape plugin. The network was enriched using CluGO plugin (Setting; Gene Ontology [GO], Molecular Function). Then, it was filtered in the CluGO plugin (GO hierarchy levels, 5–15) in other to limit the gene specificity on the GO‐Molecular Function. The network genes were subjects for the prediction of involved pathways and, the molecular events in Reactome database (https://reactome.org).

### 2.3 RT‐qPCR technique

Total RNA was extracted using GeneAll‐Hybrid‐R RNA purification kit (Cat. No. 305–101, Seoul, South Korea). Then, cDNA was synthesized using SMOBIO kit (Hsinchu, Taiwan). The BARR2 gene expression levels were measured by RT‐qPCR technique (Applied Biosystems, Foster City, CA, USA) and, were normalized with GAPDH gene expression levels. The applied primers are shown in Table 1.

**TABLE 1:** Primer sequences.

Gene	Forward primer	Reverse primer
GAPDH	CATGAGAAGTATGACAACAGCCT	AGTCCTTCCACGATACCAAAGT
BARR2	GTCAAGGTGAAGCTGGTGGT	ATGAGGTTGGTGTCCACAGG

### 2.4 Western blot technique

Total protein was extracted by RIPA buffer (Cat. No. sc‐24,948, Santa Cruz Biotechnology, USA) from the cell groups. Then, the protein samples together with prestained Protein Ladder (SL7001, SinaClon, Iran) were run on SDS‐PAGE with 8‐well comb (#1704463, BIO‐RAD) and, were transferred on PVDF membrane (IPVH00010, Merck Millipore, Darmstadt, Germany). The primary (#3857, 1:1000, Cell Signalling Technology) and conjugated secondary antibodies (#7074, 1:10000, Cell Signalling Technology) were used to detect BARR2. The protein blot was visualized with the ECL reagents (RPN2235, Amersham Biosciences, Italy) and, was quantified by ImageJ software. Data were normalized with beta‐actin (#4967, Cell Signalling Technology).

### 2.5 Statistical analysis

Graphpad Prism (version 8.0.3) was used to analyse the study data. Initially, the Kolmogorov–Simonov test was used to assess the data parametric distribution. Then, the data were compared using ANOVA. The BARR2 gene expression levels were calculated using 2‐ΔΔCT formula. The data are presented in mean and standard deviation (mean ± SD). A *p*‐value <.05 was estimated to be significant.

## 3 RESULTS

### 3.1 Gene network found many cellular signalling axes related to β‐arrestin 2

**FIGURE 1:**
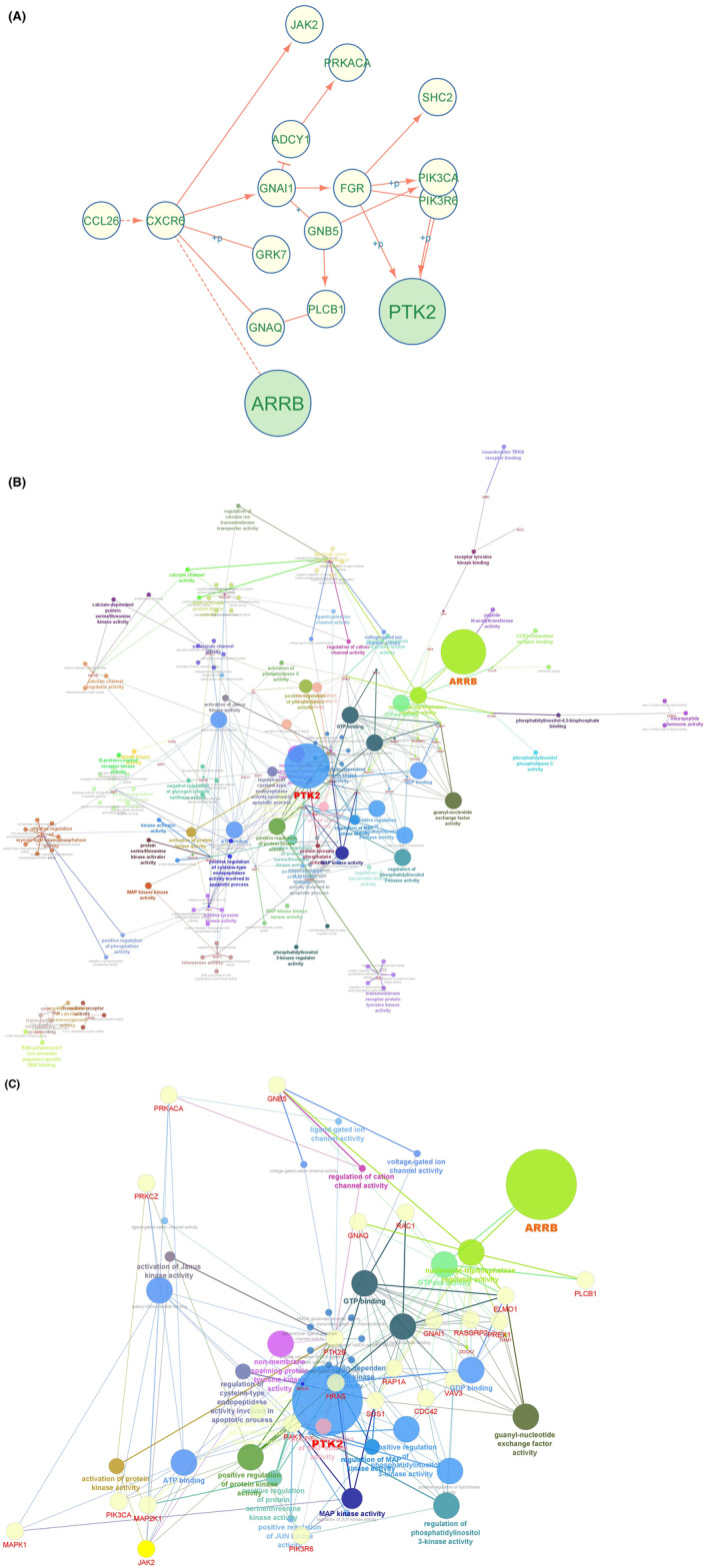
Gene networking. (A) The ARRB (BARR) and PTK2 (FAK) genes in the upstream of chemokine signalling pathway. (B) The enrichment of genes in chemokine signalling pathway using molecular function (GO, Levels 5–15). (C) The filtration of chemokine signalling pathway based on the presence of ARRB and PTK2 genes in the enriched gene network.

The BARR2 and FAK are contributed in the upstream of chemokine signalling pathway to transduce the cellular signals (Figure 1A). The functions of network genes involved in the chemokine signalling pathway are shown in Figure 1B. The filtration of gene network showed that the FAK and ARRB act highly via nucleoside triphosphate‐involved processes in the chemokine signalling pathway (Figure 1C). The Reactome data were annotated to the filtered genes so that there were many biological axes involved with the ARRB2 and FAK and their neighbour genes (Table 2).

**TABLE 2:** Prediction of Reactome signalling axes on the filtered gene network.

Reactome term ID	Description	E‐ Value
HSA‐109582	Haemostasis	5.89E‐23
HSA‐76002	Platelet activation, signalling and aggregation	4.41E‐20
HSA‐9006934	Signalling by receptor tyrosine kinases	2.37E‐19
HSA‐4420097	VEGFA‐VEGFR2 pathway	7.22E‐18
HSA‐162582	Signal transduction	8.76E‐18
HSA‐388396	GPCR downstream signalling	6.94E‐14
HSA‐5683057	MAPK family signalling cascades	6.94E‐14
HSA‐168249	Innate immune system	5.13E‐13
HSA‐2029480	Fcgamma receptor (FCGR) dependent phagocytosis	1.40E‐12
HSA‐114604	GPVI‐mediated activation cascade	1.96E‐11
HSA‐422475	Axon guidance	2.90E‐11
HSA‐166520	Signalling by NTRKs	4.05E‐11
HSA‐168256	Immune system	4.11E‐11
HSA‐3928662	EPHB‐mediated forward signalling	5.37E‐11
HSA‐2682334	EPH‐ephrin signalling	1.13E‐10
HSA‐9664422	FCGR3A‐mediated phagocytosis	4.58E‐10
HSA‐2029482	Regulation of actin dynamics for phagocytic cup formation	4.96E‐10
HSA‐194315	Signalling by Rho GTPases	8.56E‐10
HSA‐2454202	Fc epsilon receptor (FCERI) signalling	1.07E‐09
HSA‐2871796	FCERI mediated MAPK activation	1.07E‐09
HSA‐397795	G‐protein beta: gamma signalling	1.07E‐09
HSA‐9009391	Extra‐nuclear oestrogen signalling	1.31E‐09
HSA‐9656223	Signalling by RAF1 mutants	3.47E‐09
HSA‐6802946	Signalling by moderate kinase activity BRAF mutants	5.61E‐09
HSA‐6802955	Paradoxical activation of RAF signalling by kinase inactive BRAF	5.61E‐09
HSA‐9649948	Signalling downstream of RAS mutants	5.61E‐09
HSA‐5673001	RAF/MAP kinase cascade	7.89E‐09
HSA‐187037	Signalling by NTRK1 (TRKA)	1.85E‐08
HSA‐9006115	Signalling by NTRK2 (TRKB)	3.16E‐08
HSA‐416476	G alpha (q) signalling events	3.32E‐08
HSA‐6802952	Signalling by BRAF and RAF fusions	3.42E‐08
HSA‐2424491	DAP12 signalling	4.77E‐08
HSA‐422356	Regulation of insulin secretion	8.93E‐08
HSA‐416482	G alpha (12/13) signalling events	9.38E‐08
HSA‐6806834	Signalling by MET	9.38E‐08
HSA‐194840	Rho GTPase cycle	9.62E‐08

### 3.2 BARR2 gene expression levels reduced in VSMCs treated with metformin and dexamethasone

**FIGURE 2:**
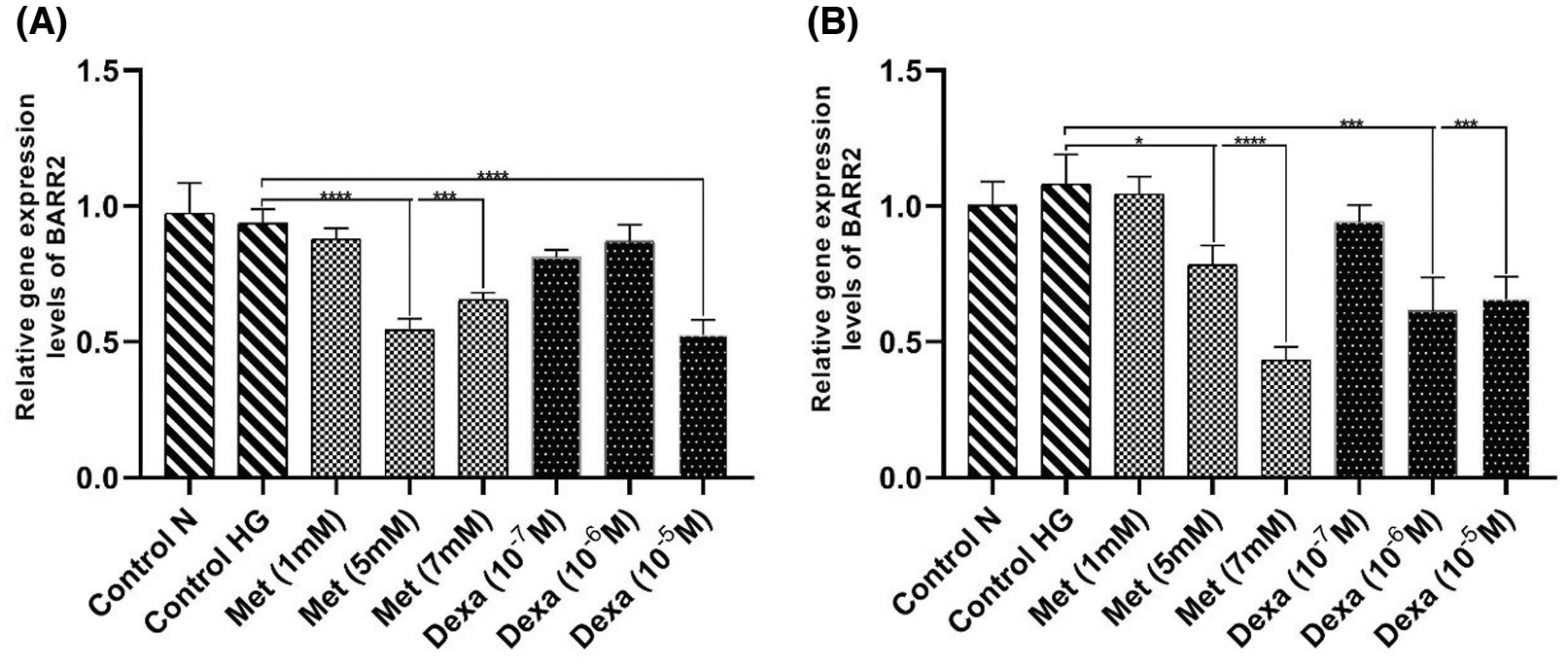
β‐arrestin 2 (BARR2) gene expression levels in vascular smooth muscle cells (VSMCs) treated with dexamethasone (Dexa) and metformin (Met) for 24 h and 48 h. (A) After 24 h of treatment. The BARR2 gene expression levels reduced significantly in the cell groups treated with Met (5 and 7 mM) and Dexa (10^−5^ M). (B) After 48 h. The BARR2 gene expression levels reduced in the cell groups treated with Met (5 and 7 mM) and Dexa (10−5 and 10−6 M). The tests are repeated three times (*n* = 3). The data are presented in Mean and Standard deviation (mean ± SD). *p < .05; **p < .001; ***p < .0001. HG, high glucose.

The BARR2 gene expression levels reduced significantly in the cell (VSMCs) groups treated with Met (1 mM, 5 mM and 7 mM) after 24 and 48 h as compared to the HG control group. However, more effects were observed in 48‐h period. Also, the BARR2 gene expression levels reduced significantly in the cell (VSMCs) groups treated with Dexa (10^−5^ M and 10^−6^ M) after 48 h. Only the group treated with Dexa 10^−5^ M reduced significantly the BARR2 gene expression levels after 24 h (*p* < .0001) (Figure 2).

### 3.3 BARR2 protein expression levels reduced in VSMCs treated with metformin and dexamethasone

**FIGURE 3:**
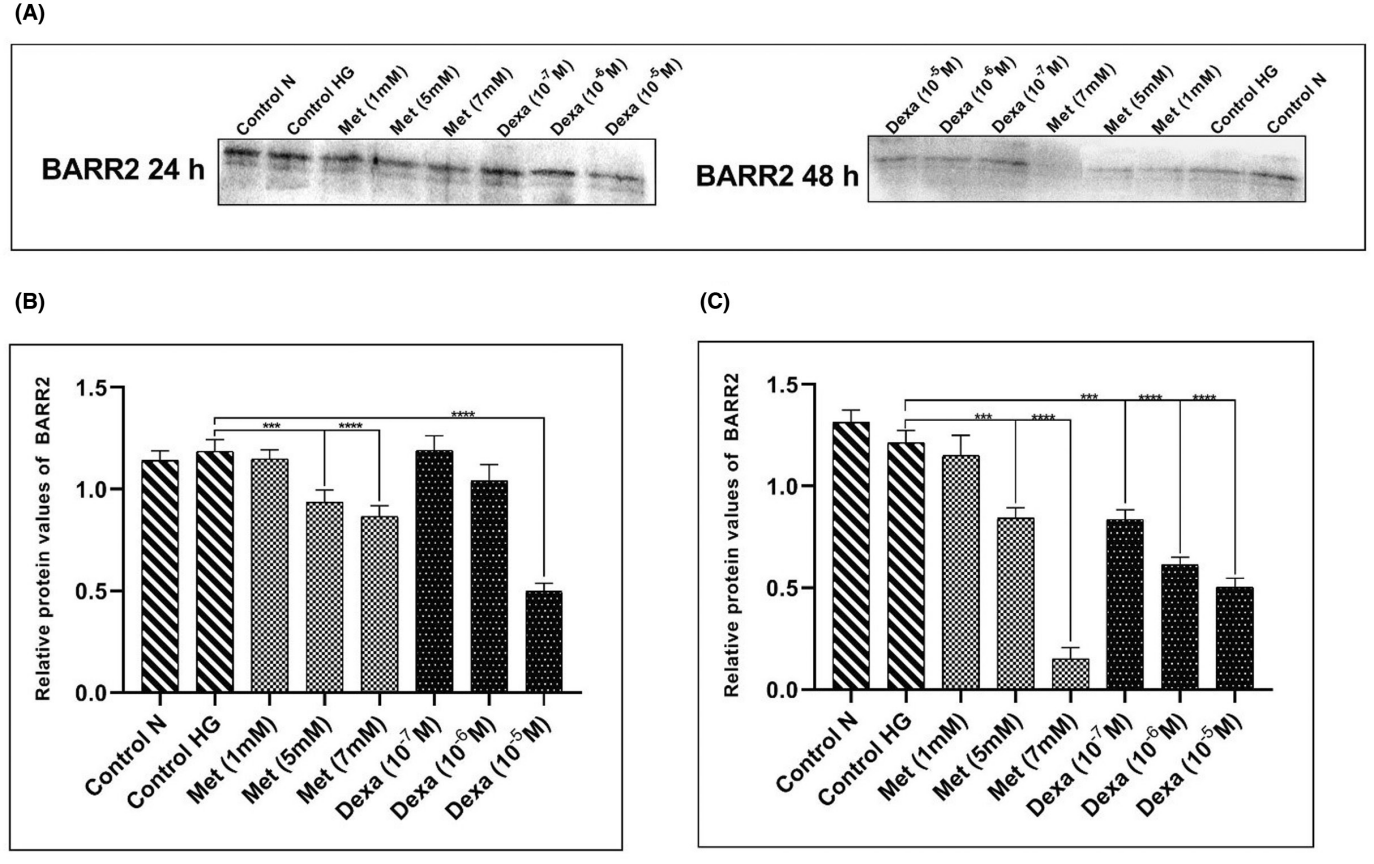
β‐arrestin 2 (BARR2) protein expression levels in vascular smooth muscle cells (VSMCs) treated with dexamethasone (Dexa) and metformin (Met) for 24 h and 48 h. (A) Western blot image. (B) After 24 h of treatment. The BARR2 protein expression levels reduced significantly in the VSMC groups treated with Met (5 and 7 mM) and Dexa (10^−5^ M). (C) After 48 h. The BARR2 protein expression levels reduced in the all of cell groups treated with Dexa (10^−5^ and 10^−6^ and 10^−7^ M) and Met (5 and 7 mM). The tests are repeated three times (*n* = 3). The data are presented in Mean and Standard deviation (mean ± SD). *p < .05; **p < .001; ***p < .0001. HG, high glucose.

The BARR2 protein expression levels decreased in VSMCs treated with Met and Dexa. In the 24‐h period, the BARR2 protein expression levels decreased significantly with values of Met 5 mM and 7 mM (*p* < .001), and Dexa 10^−5^ M (*p* < .0001). Furthermore, BARR2 protein expression levels decreased significantly in the cell groups treated with Met (5 mM and 7 mM, *p* < .001) and Dexa (10^−5^ M, 10^−6^ M, 10^−7^ M, *p* < .001) in the 48‐h period (Figure 3; Figure S1).

## 4 DISCUSSION

It is widely known that the proliferation and migration of VSMCs contribute in the progression of atherogenic plaques in the media layer of arteries.^20^, ^21^, ^22^ Furthermore, some studies have shown that VSMCs involve in the stability and rupture of atherosclerotic plaques, and finally vessel stenosis.^23^ Some studies reported that the cellular growth factors and cytokines could affect the proliferation and migration of pathways in VSMCs.^5^, ^24^ Moreover, hyperglycaemia is reported to be involved in the progression of atherosclerosis in diabetics. Other biological factors such as the activation of protein kinase C, the formation of advanced glycation end products (AGEs), and the stimulation of the lipoxygenase synthesis are suggested to relate to cardiovascular diseases.^7^, ^25^ Some studies reported that the HG‐activated STAT3/Pim‐1 signalling pathway mediates the VSMC proliferation and migration in HG conditions.^25^ Another study also reported that FAM3B stimulates the VSMC proliferation and migration in response to HG conditions.^26^ In contrast with the above studies, the VSMC proliferation and migration were not observed in HG conditions in a study, so that it proposed other subendothelial cells and modified compounds related to high blood sugar might be essential to improve the VSMC proliferation and migration.^27^

BARR2, as a tumour suppressor, suppresses the mitogenic signalling pathway in prostate cancer cells. BARR2 also inhibits Src activation by CXCR7, resulting in the inhibition of EGFR‐ and ERK‐related signalling pathways.^28^ In addition, BARR2−/− mice revealed that VSMCs have lower proliferation than the wild type. These reports suggested that the inhibition of BARR2 might suppress the progression of atherosclerosis and vessel restenosis.^10^

Met is widely used in type 2 diabetes and other disorders related to insulin resistance. Met affects oxidative stress, insulin sensitivity, gluconeogenesis, glucose absorption and consumption in the cells.^12^, ^29^ Met may slow the progression of diabetes‐related atherosclerosis by reducing VSMC proliferation and migration processes via mediating the AMPK pathway.^13^ There is also evidence that Met inhibits vascular calcification via AMPK/eNOS/NO axis.^11^ Dexa also has different biological effects, the most notable of which are the control and inhibition of immunological events. Several studies have demonstrated that Dexa can influence the function of VSMCs.^14^, ^17^, ^18^ These reports found that raising the expression of PGC‐1^18^ and modifying the activity of matrix metalloproteinase inhibit the proliferation and migration of VSMCs.^17^ The results of this study showed that Met and Dexa suppress the BARR2 protein and gene expression levels. Since BARR2 is known to involve in cell growth,^28^ thus the study suggested that Met and Dexa might inhibit the VSMC proliferation and migration. The study also suggested the cellular events related to BARR2 might be involved in many signalling axes.

## 5 CONCLUSION

The results showed that Met similar to Dexa suppresses the BARR2 protein and gene expression levels in the VSMCs. Moreover, the gene network showed that the BARR2 is a central gene to transduce the signalling messages in the chemokine signalling pathway so that the decrease of BARR2 expression level suppresses the chemokine signalling pathway via the nucleoside triphosphate metabolism as shown in the enrichment of gene network. Furthermore, the gene network showed that the BARR2‐related signalling axes might affect many cellular signalling pathways. The prediction results suggested that the decrease of BARR2 by Met and Dexa might affect the chemokine signalling pathway and other BARR2‐related signalling axes. However, more studies are needed to clear the roles of Met and Dexa in these predicted pathways.

## AUTHOR CONTRIBUTIONS

Mohammad Najafi and Ali Akbar Soleimani designed the study. Mohammad Elahimanesh predicted the gene networks. Nafiseh Shokri, Payam Mohammadi and Najmeh Parvaz evaluated the gene and protein expression levels. Masoomeh Bakhshandeh helped in the revision.

## FUNDING INFORMATION

The part of work was supported by IUMS [grant numbers 19957].

## CONFLICT OF INTEREST STATEMENT

The authors declare no conflicts of interest.

## ETHICS STATEMENT

This experiment was approved by the Ethics Committee of Iran University of Medical Sciences with the following ethical code: IR.IUMS.FMD.REC.1400.488.

## CONSENT

Informed consent was not obtained since the study was investigated on cell line.

## Supporting information


Figure S1:

## Data Availability

The data underlying this article are available from the corresponding author upon request.
