# Supplementary material for: Beta arrestin‐related signalling axes are influenced by dexamethasone and metformin in vascular smooth muscle cells cultured in high glucose condition
Source: Endocrinol Diabetes Metab. 2023 Dec 15;7(1):e465. doi: 10.1002/edm2.465 (PMC10782052; doi:10.1002/edm2.465)
Supplement: Supplementary file 1 — Figure S1: [file EDM2-7-e465-s001.docx]

**Supplementary File**

Additional file 1.

***A, 24-hour period***


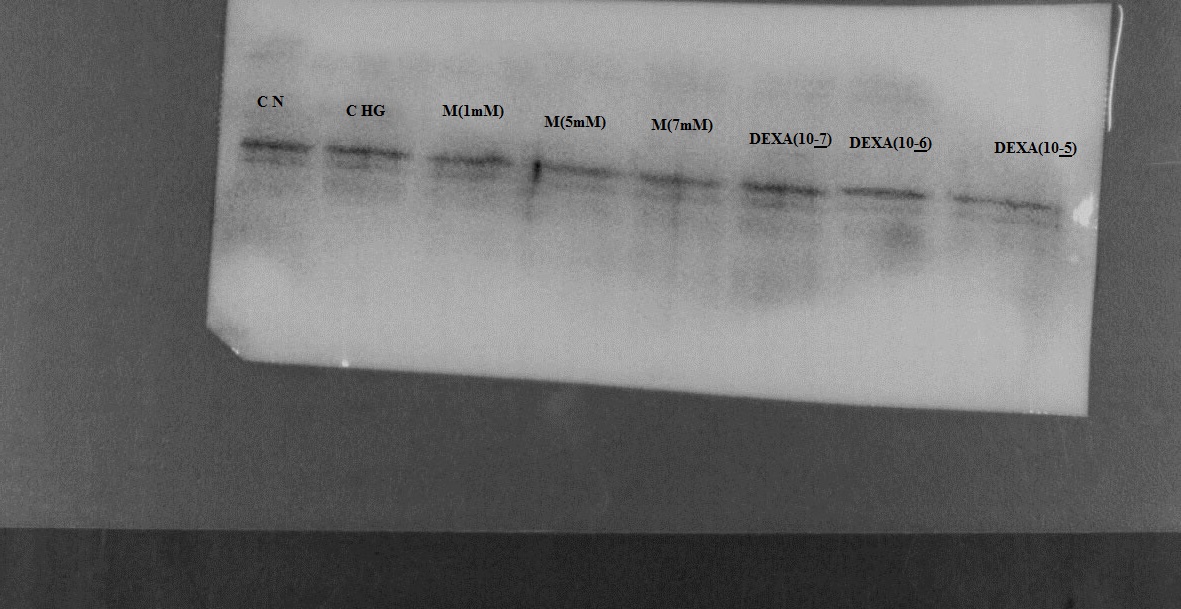


***B, 48-hour period***


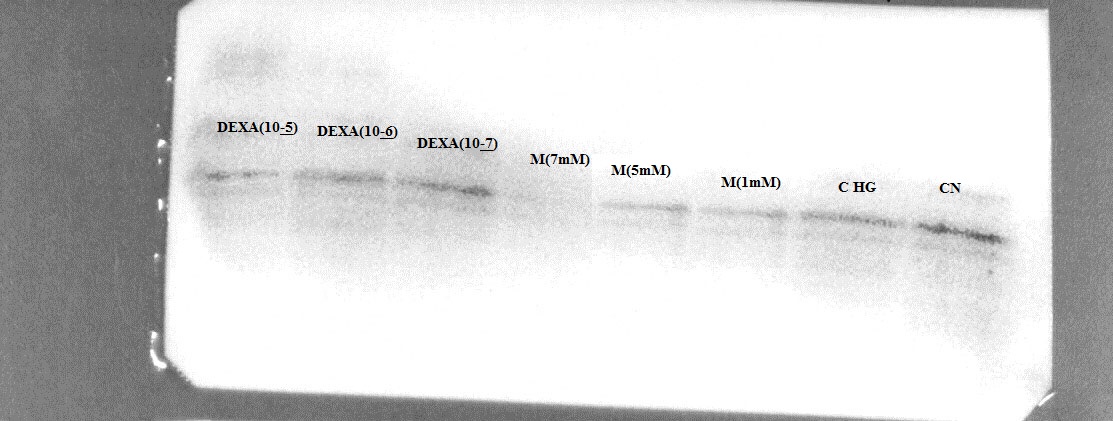


**Figure S1**: Full-size uncropped western blot images in Figure 3A. **A**, 24-hour period. **B**, 48-hour period. CN, Control Normal; CHG, Control high glucose; M, Metformin; DEXA, Dexamethasone
